# Supplementary material for: Targeted degradation of extracellular mitochondrial aspartyl-tRNA synthetase modulates immune responses
Source: Nat Commun. 2024 Jul 22;15:6172. doi: 10.1038/s41467-024-50031-7 (PMC11263397; doi:10.1038/s41467-024-50031-7)
Supplement: Supplementary file 1 — Supplementary Information [file 41467_2024_50031_MOESM1_ESM.pdf]

Targeted degradation of extracellular mitochondrial aspartyl-tRNA synthetase modulates immune responses in bacterial pneumonia.

Supplemental Figures

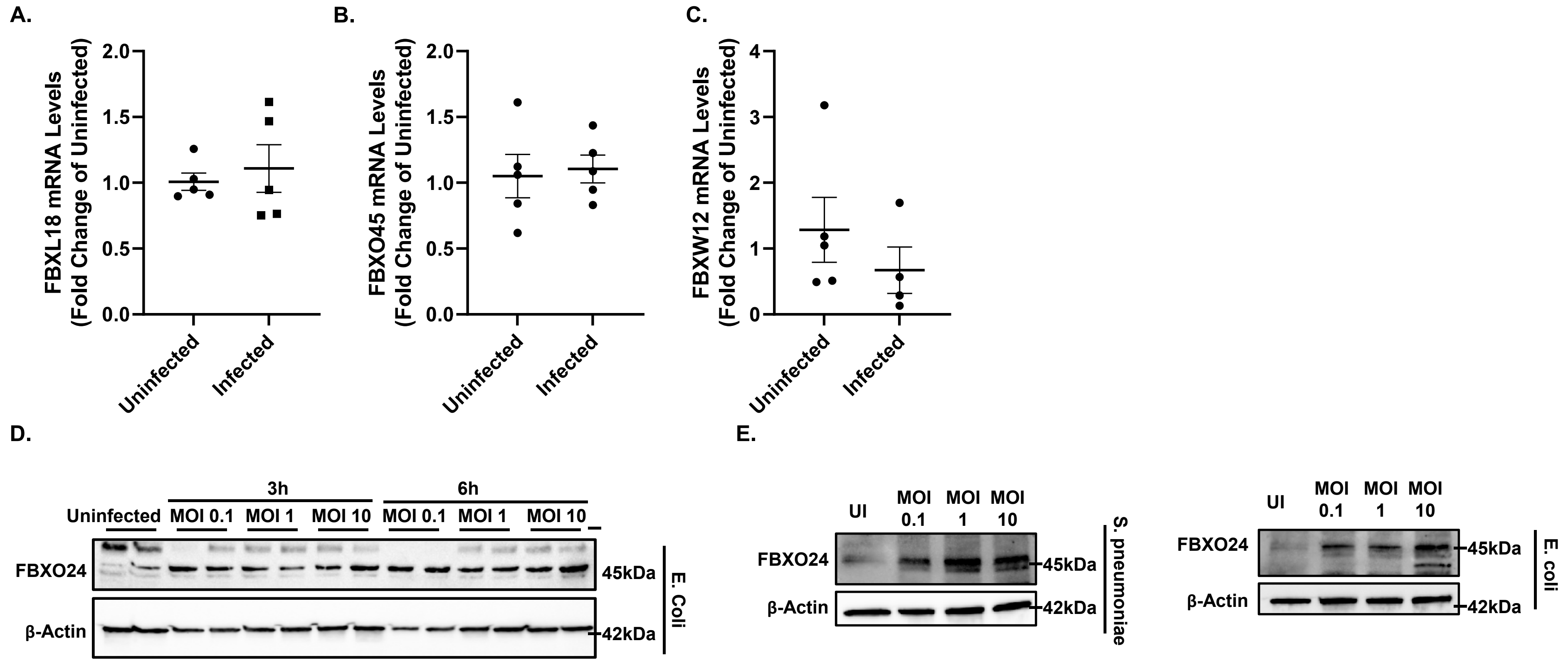

Supplementary Fig. 1. mRNA expression of (**A**) FBXL18 (**B**) FBXO45 and (**C**) FBXW12 in control or infected transplant rejected human lung tissue by RT-qPCR (*n*=5/group). (**D**) FBXO24 protein after infection of BEAS-2B cells with *E. coli* at 3 or 6h. (**E**) FBXO24 protein levels in THP-1 cells after infection with *S. pneumoniae, or E. coli* for 3h. (**A-C**) Data presented as Mean +/- SEM.

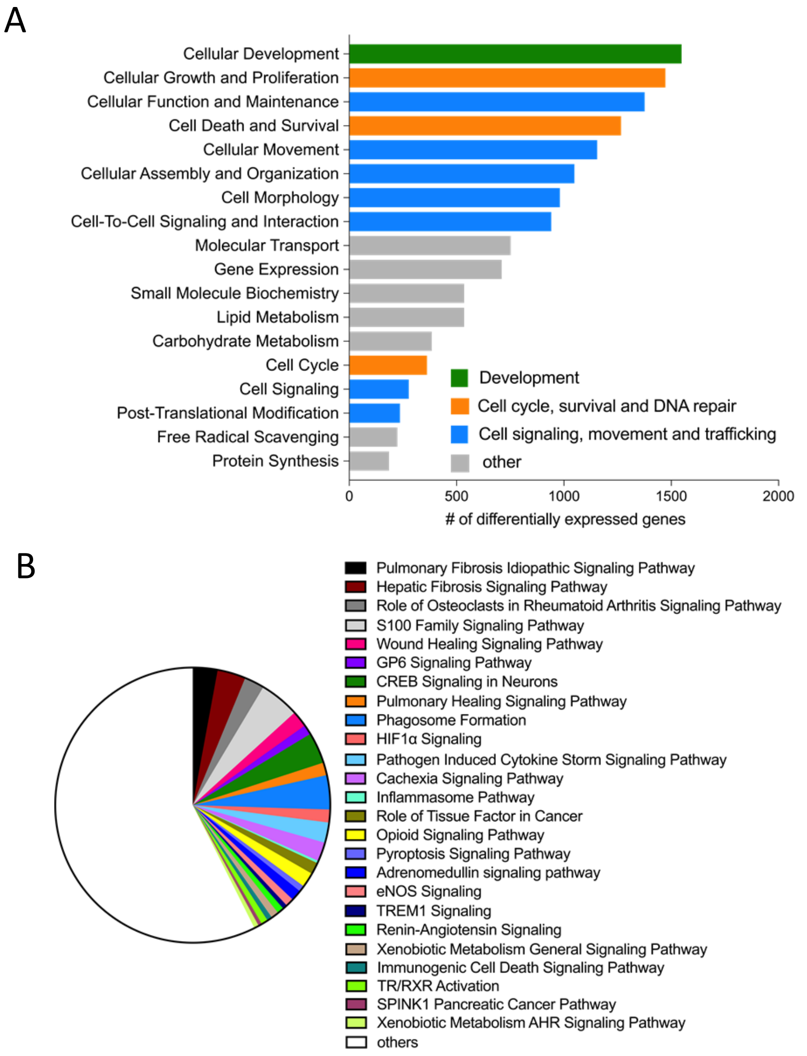

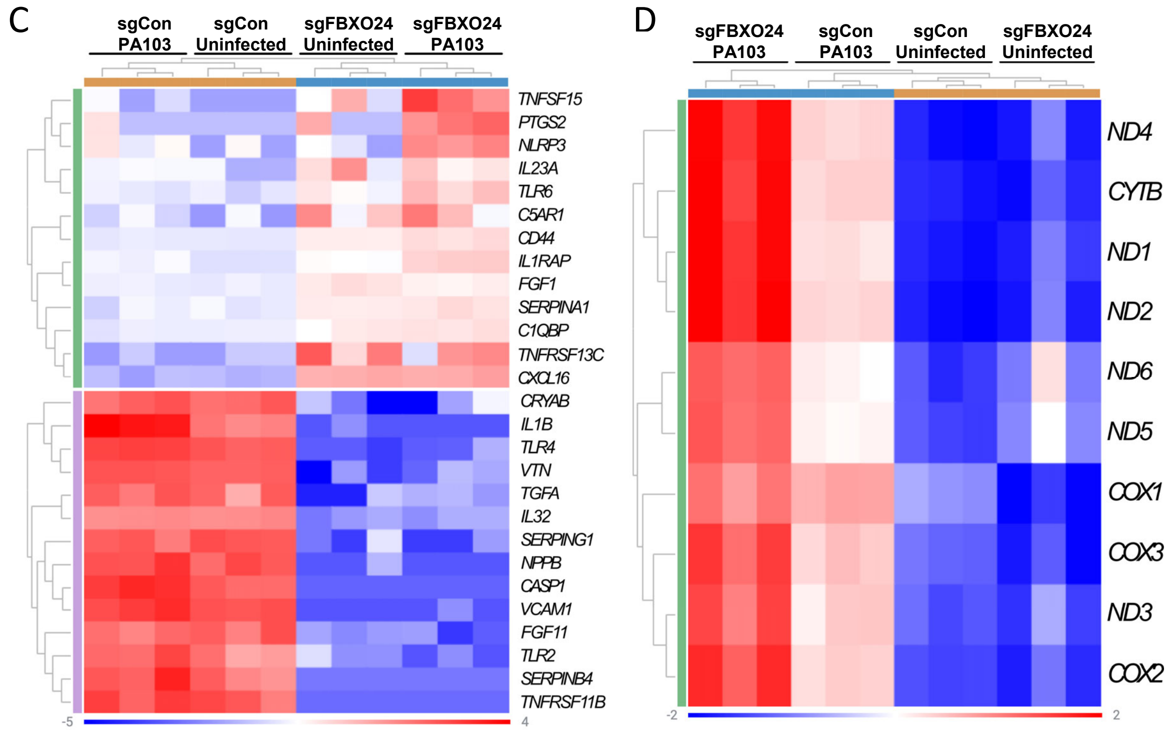

Supplemental Fig. 2. Bulk mRNA sequencing data from BEAS-2B cells following CRISPR/Cas9 knockout of *FBXO24* and infection with PA103. Analysis was done with a 1.5 fold cut-off and adjusted *P* value <0.05.
(**A**) Ingenuity pathway analysis of RNA sequencing data in uninfected BEAS-2B with CRISPR/Cas9 knockout of *FBXO24* vs. control sgRNA. Diagram shows the regulated cell function terms with the number of differentially expressed genes (DEGs) in the RNAseq dataset within each category. DEG terms were labeled according to their relevance for development (green), cell cycle, survival and DNA repair (orange), cell signaling, movement and trafficking (blue), and others (grey). (**B**) Ingenuity pathway analysis of the most regulated signaling pathways showing as % of total DEGs (absolute Z score cut-off |2.5|) for *FBXO24* sgRNA vs. control sgRNA in uninfected BEAS-2B. (**C**) Clustered heatmap demonstrates dysregulation of inflammatory genes following sgRNA for *FBXO24*. (**D**) Clustered heatmap shows upregulation of genes of the mitochondrial electron transport with PA103 infection, which is further promoted by FBXO24 knockout. Heatmap data is normalized log_2 fold expression.

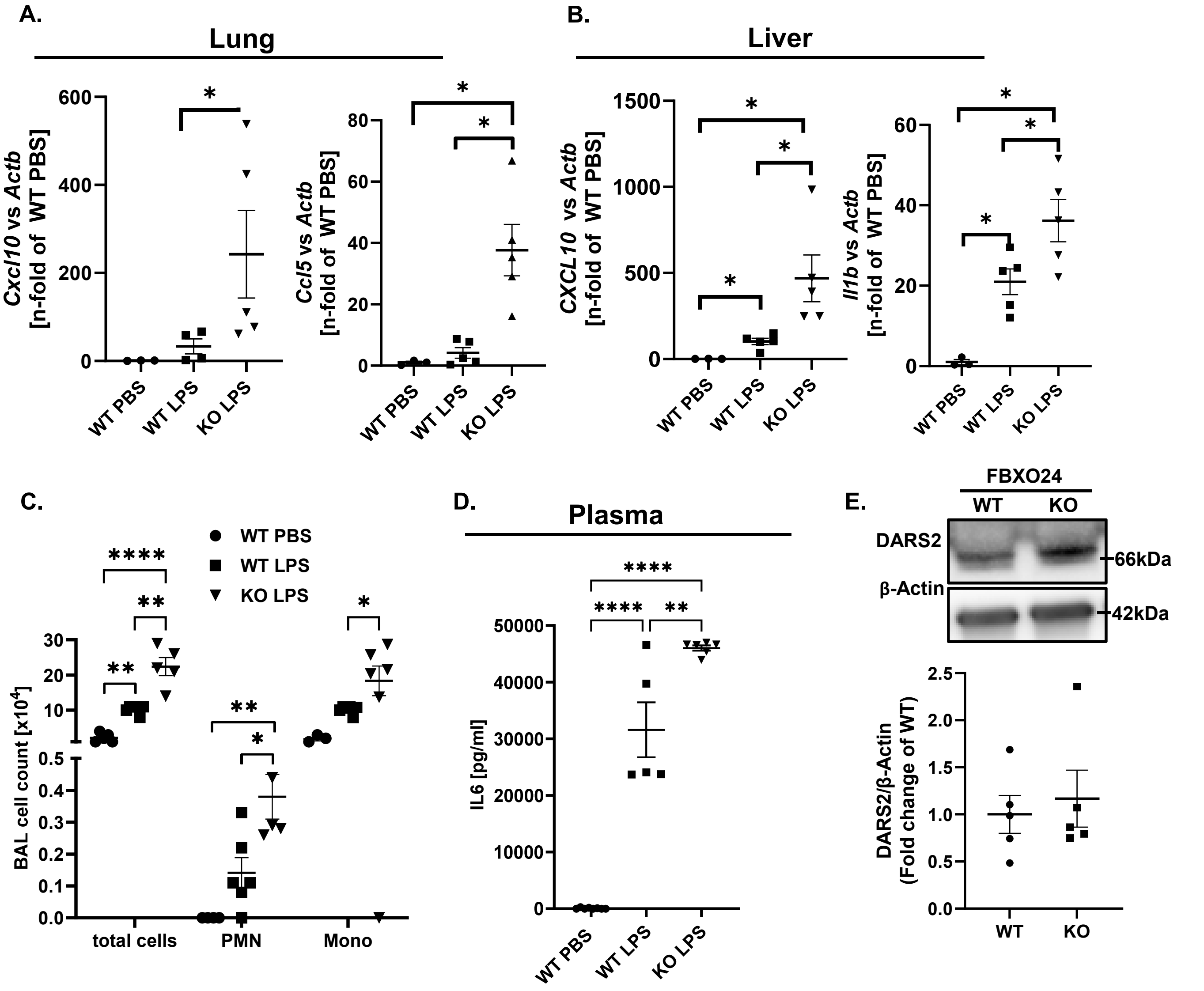

Supplemental Fig. 3. (A-C) Wild-type (WT) littermates or Fbxo24 KO mice (*n*=3-6/group) were injected i.p. with vehicle (PBS) or LPS (20mg/kg). 4 h post injection mice were euthanized, BAL and plasma were collected and liver and lung tissues harvested. (A) Lung and (B) liver tissue chemokine/cytokine mRNA expression via RT-qPCR in LPS treated or control mice. (C) BAL cell counts (Total cells from top p<0.0001, p=0.0001, p=0.0037; PMN from top p=0.0011, p=0.0149, Mono p=0.0459). (D) Plasma cytokine concentrations via multiplex ELISA in vehicle or LPS treated mice (**** p<0.0001, ** p=0.001). (E) Immunoreactive DARS2 protein levels in lung homogenates from WT littermates or Fbxo24 KO mice (*n*=5/group). Representative images and quantification by densitometry. (**A-E**) Data presented as Mean +/- SEM, p-values from Tukey's multiple comparisons.

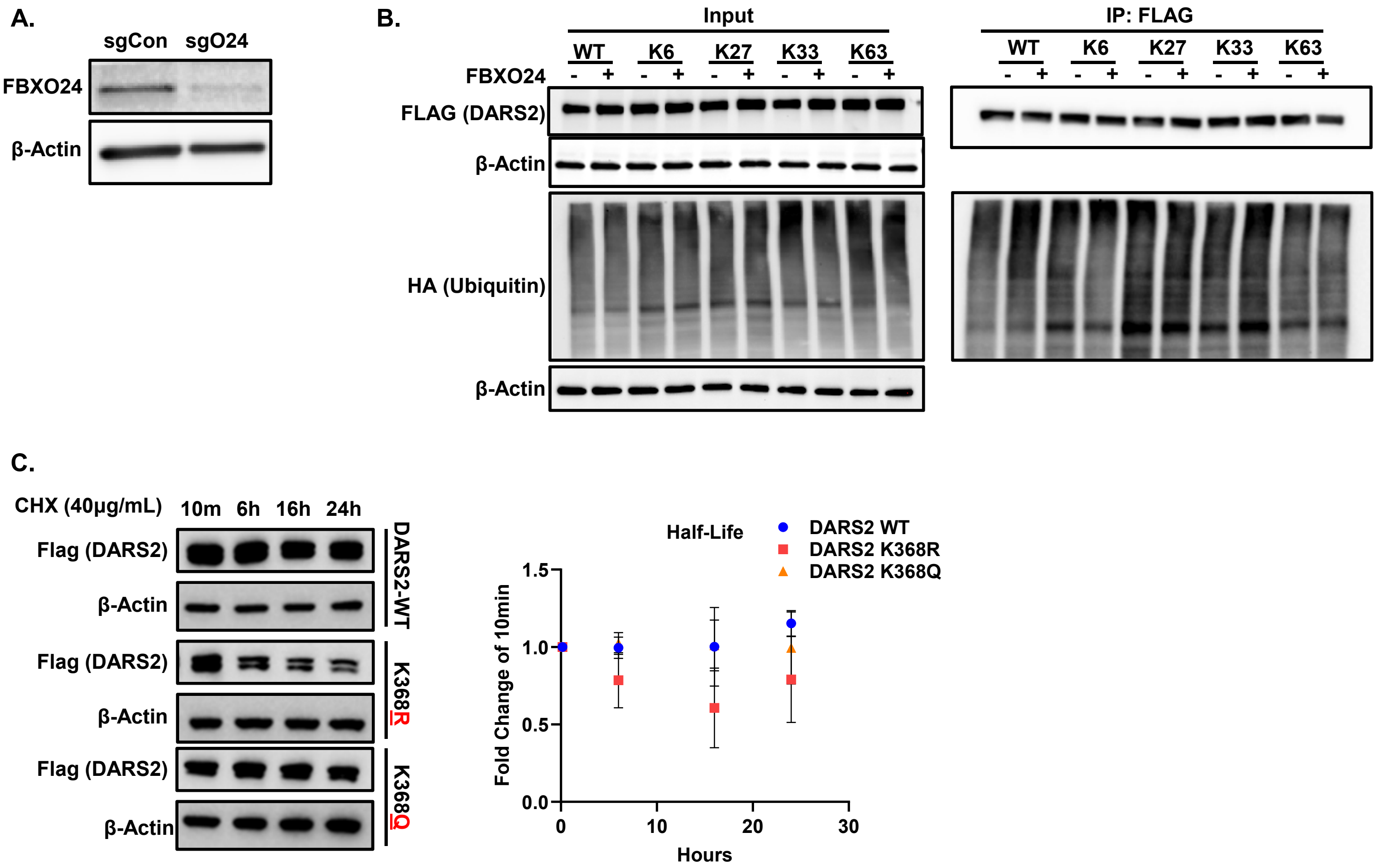

Supplementary Fig. 4. (A) Validation of FBXO24 knockdown in CRISPR SgCon versus sgO24 BEAS-2B cells. **(B)** Flag-*DARS2* co-transfected with WT or R->K HA-*ubiquitin* constructs and V5-*FBXO24* or empty vector plasmid to identify ubiquitin linkages modified by FBXO24 (*n*=2). **(C)** Ectopically expressed *DARS2*-WT, *DARS*- K368R and acetylation mimic *DARS*-K368Q in HEK293T cells treated with CHX (40µg/mL) for 10 min to 24h. Representative images and quantification of decay by densitometry are shown (*n*=3), data are presented as Mean +/- SEM.

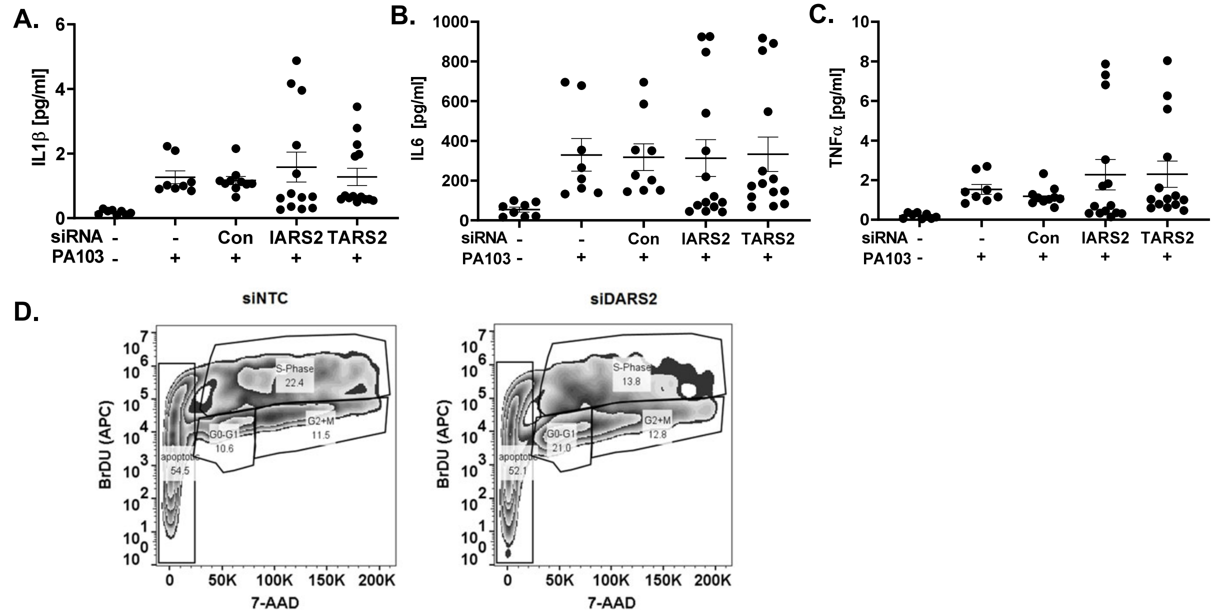

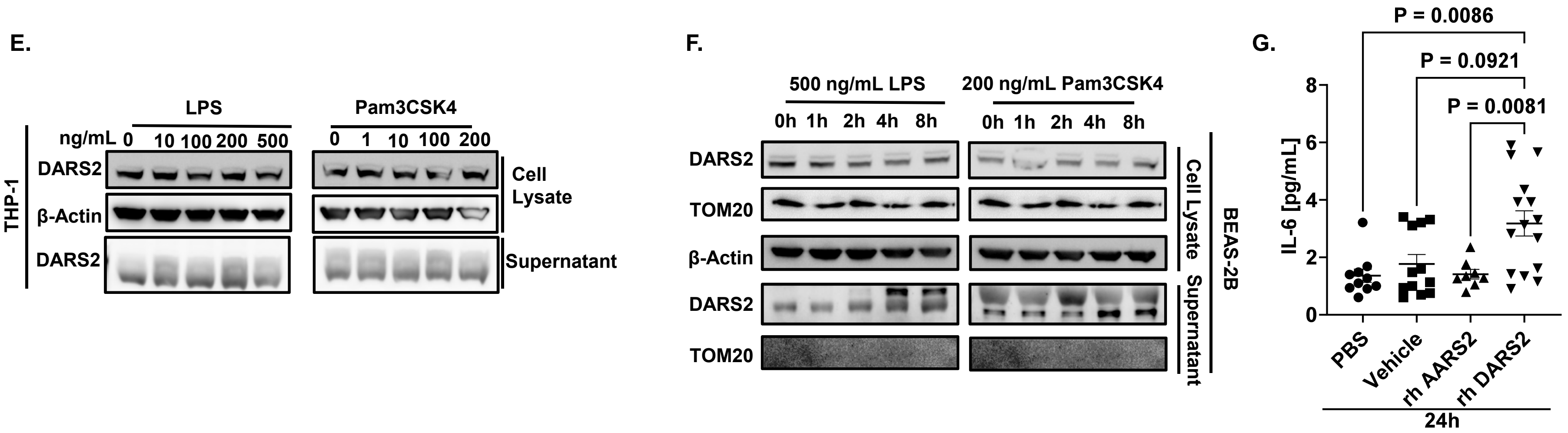

Supplementary Fig. 5. **(A-C)** BEAS-2B were transfected with control (Con) RNA, or siRNA targeting other mt-aaRS members for 72 h, then infected with PA103 at a MOI 10 for 6h; supernatant was collected and assayed for Il-6(n=3). **(D)** Gating strategy and representative image of BEAS-2B treated with siRNA scramble control or against DARS2 and stained with BrDU for cell cycle analysis from figure 6F **(E)** Effect of increasing concentrations of LPS (10-500ng/mL) or Pam3CSK4 (1-200ng/mL) on secretion of DARS2 from THP-1 cells into supernatant, representative images (*n*=3). (**F**) Time course (0-8h) of DARS2 secretion from BEAS-2B stimulated with LPS (500ng/mL) or Pam3CSK4 (200ng/mL), representative images (*n*=3). (**G**) IL-6 concentration in BAL of mice instilled i.t. with vehicle (protein transfection reagent), or recombinant DARS2 (5μg) or AARS2 (5μg) packaged in lipid vesicles for analysis at 24h (*n*=5-9/group). (**A-C, G**) Data presented as Mean +/- SEM. (**G**) P-Values derived from Brown-Forsythe and Welch ANOVA tests with Dunnet's T3 multiple comparisons.

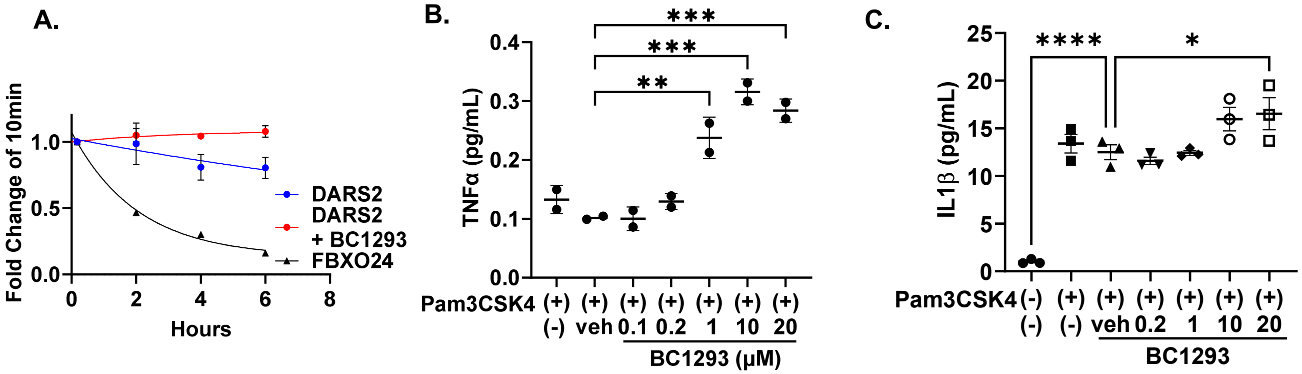

Supplementary Fig. 6. (A) DARS2 half-life extended by BC-1293 from quantification of Fig.7f. **(B)** TNFα in supernatant of BEAS-2B treated with BC-1293 or DMSO control in the absence or presence of Pam3CSK4 (1mg/mL) (n=3) (from top p=0.0003, p=0.0001, p=0.0018). (**C**) IL-1β was assayed in supernatants of primary CD14+ cells, differentiated with M-CSF, and stimulated with or without Pam3CSK4 (1mg/mL) and treated with BC-1293 or DMSO (vehicle, [veh]) control (left p<0.0001, right p= 0.0390). **(B**, **C**) analyzed with One-way Anova with Dunnett's multiple comparisons test and data presented as Mean +/- SEM**.**

Supplementary Table 1.Clinical Features of Patients					
	Control (*n*=10)	Pseudomonas Negative (*n*=37)	Pseudomonas Positive (*n*=3)	Total (*n*=50)	*P* value
Age (Mean years +SD)	54.100 (5.519)	60.155 (14.493)	60.751 (17.314)	58.980 (13.351)	0.441
Male gender at birth	6 (60.0%)	22 (59.5%)	2 (66.7%)	30 (60.0%)	0.970
Caucasian	7 (70.0%)	32 (86.5%)	2 (66.7%)	41 (82.0%)	0.376
In Hospital Mortality	0 (0.0%)	19 (51.4%)	1 (33.3%)	20 (40.0%)	0.013

Supplementary Table 2. Primers used In the Study

Species	Primer	Forward	Reverse
Human	FBXO24	CCT ATT CTG TGC TCT TGG CTA C	TAA CAA ACC TGT GTG GGT TCC
Human	FBXO45	CTA CAT AAT GGA GAA GTC AAT GG	GAC CG AAT TCT TTC TCC TAT CTG
Human	FBXW12	CAT CCA AGC ATA TGA GAT CGC	AGA TTC GTA GCT GGA TGC C
Human	β-Actin	AGG CAC CAG GGC GTG AT	GCC CAC ATA GGA GTC CTT CTG AC
Mouse	IL-1β	CCT CCA AGG AAA GAA TCT ATA CCT G	CTT GGG ATC CAC ACT CTC C
Mouse	IL-6	TAC CAC TTC ACA AGT CGG A	AAT TGC CAT TGC ACA ACT C
Mouse	CXCL10	GAC TCA AGG GAT CCC TCT C	ATG GCC CTC ATT CTC ACT G
Mouse	KC	GTC GGG ATT CAC CTC AAG AA	GTG TGG CTA TGA CTT CGG TT
Mouse	G-CSF	ACC TAC AAG CTG TGT CAC C	CTT AGG CAC TGT GTC TGC T
Mouse	CCL5	TCT TGC AGT CGT GTT TGT C	GTT GAT GTA TTC TTG AAC CCA C
Mouse	β-Actin	GCT ATG TTG CTC TAG ACT TCG	GGA TTC CAT ACC CAA GAA GG
