# Supplementary material for: Targeted degradation of extracellular mitochondrial aspartyl-tRNA synthetase modulates immune responses
Source: Nat Commun. 2024 Jul 22;15:6172. doi: 10.1038/s41467-024-50031-7 (PMC11263397; doi:10.1038/s41467-024-50031-7)
Supplement: Supplementary file 3 — Description of Additional Supplementary Files [file 41467_2024_50031_MOESM3_ESM.pdf]

Description of Additional Supplementary Files

File Name: Supplementary Data 1
Description: Guide (g) and small interfering (si) RNA sequences. The top of this table lists the gRNAs used for CRISP-Cas9 generation of FBXO24-KO cell lines. The bottom portion of the table lists siRNA sequences used for transient knockdown studies.
