# Supplementary material for: Targeted degradation of extracellular mitochondrial aspartyl-tRNA synthetase modulates immune responses
Source: Nat Commun. 2024 Jul 22;15:6172. doi: 10.1038/s41467-024-50031-7 (PMC11263397; doi:10.1038/s41467-024-50031-7)
Supplement: Supplementary file 5 — Reporting Summary [file 41467_2024_50031_MOESM5_ESM.pdf]

nature portfolio

Corresponding author(s): Rama Mallampalli

Last updated by author(s): 5/30/2024

Reporting Summary

Nature Portfolio wishes to improve the reproducibility of the work that we publish. This form provides structure for consistency and transparency in reporting. For further information on Nature Portfolio policies, see our Editorial Policies and the Editorial Policy Checklist.

Statistics

For all statistical analyses, confirm that the following items are present in the figure legend, table legend, main text, or Methods section.

n/a	Confirmed	
☐	☒	The exact sample size (*n*) for each experimental group/condition, given as a discrete number and unit of measurement
☐	☒	A statement on whether measurements were taken from distinct samples or whether the same sample was measured repeatedly
☐	☒	The statistical test(s) used AND whether they are one- or two-sided<br>*Only common tests should be described solely by name; describe more complex techniques in the Methods section.*
☐	☒	A description of all covariates tested
☐	☒	A description of any assumptions or corrections, such as tests of normality and adjustment for multiple comparisons
☐	☒	A full description of the statistical parameters including central tendency (e.g. means) or other basic estimates (e.g. regression coefficient) AND variation (e.g. standard deviation) or associated estimates of uncertainty (e.g. confidence intervals)
☐	☒	For null hypothesis testing, the test statistic (e.g. *F*, *t*, *r*) with confidence intervals, effect sizes, degrees of freedom and *P* value noted<br>*Give P values as exact values whenever suitable.*
☒	☐	For Bayesian analysis, information on the choice of priors and Markov chain Monte Carlo settings
☒	☐	For hierarchical and complex designs, identification of the appropriate level for tests and full reporting of outcomes
☒	☐	Estimates of effect sizes (e.g. Cohen's *d*, Pearson's *r*), indicating how they were calculated

Our web collection on statistics for biologists contains articles on many of the points above.

Software and code

Policy information about availability of computer code

Data collection	Wave (v2.6.3), Gen5 (v3.05), Bio-Rad CFX Maestro, ImageLab (v6.0.1), ImageJ (v1.53e), Discovery Workbench (v4.0), Flexiware, Discovery studio (v4.1), NovoSeq P150
Data analysis	R (v4.2.2) , FloJo, GraphPad Prism (v10.2.0), Mascot Daemon (v2.7.0), ProteomeDiscoverer (v2.4), ImageLab (v6.0.1), ImageJ (v1.53e), Discovery Workbench (v4.0), Excel2016, Discovery studio (v4.1), ROSALIND

For manuscripts utilizing custom algorithms or software that are central to the research but not yet described in published literature, software must be made available to editors and reviewers. We strongly encourage code deposition in a community repository (e.g. GitHub). See the Nature Portfolio guidelines for submitting code & software for further information.

Data

Policy information about availability of data

All manuscripts must include a data availability statement. This statement should provide the following information, where applicable:

- Accession codes, unique identifiers, or web links for publicly available datasets
- A description of any restrictions on data availability
- For clinical datasets or third party data, please ensure that the statement adheres to our policy

Uncropped blots and data used to generate graphs are included in the source data file uploaded with this paper. Proteomic data from this study has been deposited in the ProteomeXchange database with the identifier PXD050224. RNA-seq data has been deposited in the NCBI GEO database and will be available with

the accession code GSE260733.
Proteomic data is currently available to reviewers/editors using the following login, username: reviewer_pxd050224@ebi.ac.uk and password: dBu11k8I. Upon acceptance of publication ProteomeXChange requires notification and will make data publicly available.

Research involving human participants, their data, or biological material

Policy information about studies with human participants or human data. See also policy information about sex, gender (identity/presentation), and sexual orientation and race, ethnicity and racism.

Reporting on sex and gender	Findings are not sex or gender dependent. The demographics of our samples are listed in supplemental table 1.
Reporting on race, ethnicity, or other socially relevant groupings	Socially determined variables were not include in our analysis of DARS2 levels in patient plasma. The demographics of our samples are listed in supplemental table 1.
Population characteristics	The population characteristics of human plasma samples are listed in Supplementary Table 1
Recruitment	40 patients with acute respiratory failure who were admitted to the medical intensive care unit (MICU) at the Ohio State University Wexner Medical Center and The James Cancer Hospital between May 2020 and December of 2021 were enrolled within 48 h of ICU admission. Biospecimens, including peripheral blood collected in sodium citrate tubes, are collected on days 1, 7, and 21 of ICU admission. These raw data are available in the ProteomeXchange database with the identifier PXD050224.
Ethics oversight	Ohio State University Institutional Review Board, (IRB protocol #2020H0175).

Note that full information on the approval of the study protocol must also be provided in the manuscript.

Field-specific reporting

Please select the one below that is the best fit for your research. If you are not sure, read the appropriate sections before making your selection.

☒ Life sciences ☐ Behavioural & social sciences ☐ Ecological, evolutionary & environmental sciences

For a reference copy of the document with all sections, see nature.com/documents/nr-reporting-summary-flat.pdf

Life sciences study design

All studies must disclose on these points even when the disclosure is negative.

Sample size	No statistical method was used to predetermine sample size.
Data exclusions	For animal studies outliers were removed with Data with Robust regression and Outlier removal (ROUT) method with Q=0.1% as the threshold for removal. This method was also used for In vitro studies with n>3 when appropriate.
Replication	To ensure a high level of reproducibility experiments were performed using at least n=3 of biological replicates unless otherwise noted in the figure legend or text. Many studies were also reproduced by multiple investigators. Additionally many experiments in support of the same conclusions were conducted by different experimenters; for example studies on the DARS2-K-R mutants were carried out by two separate experimenters one conducting ubiquitinylation/acetylation experiments and the other examining changes in half life. These yielded logically consistent results and offers a high level of confidence in the general validity and reproducibility of the work.
Randomization	For In Vivo experiments mice of the same genetic background were randomly assigned to experimental groups. Like wise mouse lung slices from each mouse were randomly distributed throughout treatment groups to negate animal effects. For in vitro studies randomization was not relevant as there is only one Beas2B, THP1, HEK etc cell line and this precludes the ability to randomize cells between groups.
Blinding	Blinding was used for scoring of Acute Lung injury score parameters. Blinding was done during data analysis for the lung histology. Images were received from the core blinded and then analyzed for ALI score. Blinding was also used for data Collection of IF for figure 3 B/C and O. Data was collected by one individual and then blinded to be evaluated by another. Blinding was used in these studies as these were subjective in nature. Otherwise blinding was not used as in vitro methods in this study used objective measurements which produce quantitative data. Additionally individual experimenters were responsible for conducting in vitro experiments from set up through to data collection. In this context blinding was not feasible. This is compensated for in the as described in the replication section. Similarly for other components of the In Vivo experiments were also objective/measures of cytokine concentration assayed by ELISA, cell number, mRNA by RT-qPCR, etc.

Reporting for specific materials, systems and methods

We require information from authors about some types of materials, experimental systems and methods used in many studies. Here, indicate whether each material, system or method listed is relevant to your study. If you are not sure if a list item applies to your research, read the appropriate section before selecting a response.

Materials & experimental systems

n/a	Involved in the study
☐	☒ Antibodies
☐	☒ Eukaryotic cell lines
☒	☐ Palaeontology and archaeology
☐	☒ Animals and other organisms
☐	☒ Clinical data
☒	☐ Dual use research of concern
☒	☐ Plants

Methods

n/a	Involved in the study
☒	☐ ChIP-seq
☐	☒ Flow cytometry
☒	☐ MRI-based neuroimaging

Antibodies

Antibodies used

Antibodies used in this project are listed below with their associated catalog number under the corresponding company.

Cell Signaling Technology:
Tom20, 42406
PGC-alpha, 2178S
GAPDH, 2128S
Ubiquitin (E4I2J), 43124S
FLAG-tag, DYKDDDDK Tag (D6W5B) Rabbit mAb (Binds to same epitope as Sigma-Aldrich Anti-FLAG M2 antibody), 14793S
V5-Tag, 132025S
TFAM, 7495S
Acetylated-Lysine, 9441S
CyclinD1, 2922

Protein Tech:
DARS2, 13807-1-AP
IARS2, 17170-1-AP

NOVUS:
FBXO24, NBP1-53141
HA-Tag, NB600-362

Santa Cruz Biotechnology:
Tim23, sc-514463

Sigma Aldrich:
Beta-Actin, A5441

Aviva Systems Biology:
FBXO45, OAAB19416
FBXL2, ARP43096_P050

AbNova:
FBXL18, H00080028A01

Validation

For validation statements please refer to manufacturer websites as listed below:

Cell Signaling Technology:
Tom20, https://www.cellsignal.com/products/primary-antibodies/tom20-d8t4n-rabbit-mab/42406
PGC-alpha, https://www.cellsignal.com/products/primary-antibodies/pgc-1a-3g6-rabbit-mab/2178
GAPDH, https://www.cellsignal.com/products/primary-antibodies/gapdh-14c10-rabbit-mab/2118
Ubiquitin (E4I2J), https://www.cellsignal.com/products/primary-antibodies/ubiquitin-e4i2j-rabbit-mab/43124
"FLAG-tag", DYKDDDDK Tag (D6W5B) Rabbit mAb (Binds to same epitope as Sigma-Aldrich Anti-FLAG M2 antibody), https://www.cellsignal.com/products/primary-antibodies/dykddddk-tag-d6w5b-rabbit-mab-binds-to-same-epitope-as-sigma-aldrich-anti-flag-m2-antibody/14793
V5-Tag, https://www.cellsignal.com/products/primary-antibodies/v5-tag-d3h8q-rabbit-mab/13202
TFAM, https://www.cellsignal.com/products/primary-antibodies/tfam-antibody/7495
Acetylated-Lysine, https://www.cellsignal.com/products/primary-antibodies/acetylated-lysine-antibody/9441
CyclinD1, https://www.cellsignal.com/products/primary-antibodies/cyclin-d1-antibody/2922

Protein Tech:
DARS2, https://www.ptglab.com/products/DARS2-Antibody-13807-1-AP.htm
IARS2, https://www.ptglab.com/products/IARS2-Antibody-17170-1-AP.htm

NOVUS:
FBXO24, https://www.novusbio.com/products/fbxo24-antibody_nbp1-53141#datasheet
HA-Tag, https://www.novusbio.com/products/ha-tag-antibody_nb600-362

AbNova:
FBXL18, https://www.fishersci.com/shop/products/mouse-anti-fbxl18-polyclonal-abnova/89107353

Santa Cruz Biotechnology:
Tim23, https://www.scbt.com/p/tim23-antibody-h-8?requestFrom=search

Sigma Aldrich:
Beta-Actin, https://www.sigmaaldrich.com/US/en/product/sigma/a5441

Aviva Systems Biology:
FBXO45, https://www.avivasysbio.com/fbxo45-antibody-n-terminal-region-oaab19416.html
FBXL2, https://www.avivasysbio.com/fbxl2-antibody-n-terminal-region-arp43096-p050.html

Eukaryotic cell lines

Policy information about cell lines and Sex and Gender in Research

Cell line source(s)	Human cell lines A549, BEAS-2B, HEK293T and THP1 cells were purchased from ATCC.
Authentication	We did not conduct authentication independent of information provided by ATCC
Mycoplasma contamination	cell lines were tested regularly for mycoplasma and only negative cell lines were used in experiments
Commonly misidentified lines (See ICLAC register)	No commonly misidentified cell lines were used

Animals and other research organisms

Policy information about studies involving animals; ARRIVE guidelines recommended for reporting animal research, and Sex and Gender in Research

Laboratory animals	Mus Musculus, C57B6J, aged 3-5 months.
Wild animals	No wild animals were used
Reporting on sex	No findings in this study pertain solely to one sex. Animals of both sexes were used in studies whenever possible based the distribution of our FBXO24 Wt, Heterozygous and KO mice from breeding pairs. In no studies were data collected in a sex dependent manner.
Field-collected samples	No field samples were collected.
Ethics oversight	Animal protocols and procedures were approved by the Ohio State University Institutional Animal Care and Use Committee (IACUC). Animals were house in facilities monitored by OSU University Lab Animal Resources (ULAR) staff who ensured study team compliace with the protocol and animal welfare guidelines.

Note that full information on the approval of the study protocol must also be provided in the manuscript.

Clinical data

Policy information about clinical studies

All manuscripts should comply with the ICMJE guidelines for publication of clinical research and a completed CONSORT checklist must be included with all submissions.

Clinical trial registration	*Provide the trial registration number from ClinicalTrials.gov or an equivalent agency.*
Study protocol	*Note where the full trial protocol can be accessed OR if not available, explain why.*
Data collection	*Describe the settings and locales of data collection, noting the time periods of recruitment and data collection.*
Outcomes	*Describe how you pre-defined primary and secondary outcome measures and how you assessed these measures.*

Plants

Seed stocks

Report on the source of all seed stocks or other plant material used. If applicable, state the seed stock centre and catalogue number. If plant specimens were collected from the field, describe the collection location, date and sampling procedures.

Novel plant genotypes

Describe the methods by which all novel plant genotypes were produced. This includes those generated by transgenic approaches, gene editing, chemical/radiation-based mutagenesis and hybridization. For transgenic lines, describe the transformation method, the number of independent lines analyzed and the generation upon which experiments were performed. For gene-edited lines, describe the editor used, the endogenous sequence targeted for editing, the targeting guide RNA sequence (if applicable) and how the editor was applied.

Authentication

Describe any authentication procedures for each seed stock used or novel genotype generated. Describe any experiments used to assess the effect of a mutation and, where applicable, how potential secondary effects (e.g. second site T-DNA insertions, mosiacism, off-target gene editing) were examined.

Flow Cytometry

Plots

Confirm that:

☒ The axis labels state the marker and fluorochrome used (e.g. CD4-FITC).

☒ The axis scales are clearly visible. Include numbers along axes only for bottom left plot of group (a 'group' is an analysis of identical markers).

☒ All plots are contour plots with outliers or pseudocolor plots.

☒ A numerical value for number of cells or percentage (with statistics) is provided.

Methodology

Sample preparation

Cells were then collected, fixed, permeabilized, stained and intensity was assayed by flow cytometry using the APC BrdU Flow Kit (BD Pharmigin) according to the manufacturer’s instructions.

Instrument

BD FACSSymphony A1 (BD Bioscience)

Software

FlowJo (FlowJo, LLC).

Cell population abundance

individual cell populations were not measured. Flow cytometry was used for cell cycle progression assay, from figure 6f, using using the APC BrdU Flow Kit (BD Pharmigin)

Gating strategy

Recorded cells were graphed first on SSC/FSC (linear scale). Gate was set to remove cells on the extreme edges of X- and Y-axis border. Next, cells were graphed and gated to exclude doublets, using X-axis FSC-H and Y-axis FSC-A (both in linear scale). Then, cells were graphed according to manufactures instructions: 7-AAD (DNA stain) was graphed on X-Axis in linear scale; APC for BrdU stain was graphed on Y-Axis in log scale. Populations for S-phase (APC positive gate, 7-AAD low and high; horseshoe pattern), G0 (APC negative, low 7-AAD content) and G2+M gate (APC negative, high 7-AAD content).

☒ Tick this box to confirm that a figure exemplifying the gating strategy is provided in the Supplementary Information.
